# Imaging Assessment of Complex Renal Cysts: Comparative Value of Superb Microvascular Imaging and Contrast-Enhanced Ultrasound

**DOI:** 10.3390/jpm16010033

**Published:** 2026-01-05

**Authors:** Fabrizio Urraro, Nicoletta Giordano, Vittorio Patanè, Roberto Calbi, Alfredo Clemente, Maria Chiara Brunese, Salvatore Cappabianca, Alfonso Reginelli

**Affiliations:** 1 Department of Life Sciences, Health and Health Professions, Link Campus University, 00165 Rome, Italy; f.urraro@unilink.it (F.U.); a.clemente@unilink.it (A.C.); 2 Department of Precision Medicine, University of Campania “Luigi Vanvitelli”, Piazza Luigi Miraglia 2, 80138 Naples, Italy; 3 Department of Radiology, “F. Miulli” General Hospital, Acquaviva delle Fonti, 70021 Bari, Italy; r.calbi@miulli.it; 4 Department of Medicine and Health Sciences “Vincenzo Tiberio”, University of Molise, 86100 Campobasso, Italy

**Keywords:** Bosniak classification, Contrast-Enhanced Ultrasound (CEUS), diagnostic accuracy, reproducibility, renal cysts, Superb Microvascular Imaging (SMI)

## Abstract

**Background**: Accurate characterization of complex renal cystic lesions is essential for individualized patient management, as enhancement patterns of septa and walls determine Bosniak classification, malignancy risk, and tailored follow-up strategies. While contrast-enhanced ultrasound (CEUS) is widely used to assess enhancement, Superb Microvascular Imaging (SMI) offers a non-contrast alternative that is capable of detecting slow-flow microvascular signals. This study aimed to evaluate the diagnostic concordance, accuracy, and reproducibility of SMI compared with CEUS in the Bosniak 2019 classification, and to explore its role in personalized imaging pathways for patients with contraindications to contrast media. **Methods**: Eighty patients (92 cystic renal lesions) who underwent both SMI and CEUS between January 2024 and July 2025 were retrospectively analyzed. Lesions were categorized using the Bosniak 2019 criteria. CEUS served as the reference standard. Concordance between modalities was evaluated using Cohen’s κ, and diagnostic accuracy was determined by ROC analysis. Inter- and intra-reader agreement were assessed with κ and intraclass correlation coefficients (ICC), respectively. Histopathologic confirmation was available for resected Bosniak III–IV lesions. **Results**: SMI showed excellent concordance with CEUS (κ = 0.84, 95% CI 0.76–0.91; overall agreement 83.7%). Concordance was perfect for Bosniak I–II, good for IIF (85%), and moderate for III (68%) and IV (64%) categories. Using CEUS as the reference, SMI achieved a sensitivity of 88.5%, specificity of 90.0%, and AUC of 0.94 for distinguishing low- from high-risk lesions. Inter-reader (κ = 0.83) and intra-reader (ICC = 0.91) agreements were excellent. Among 18 surgically resected Bosniak III–IV lesions, 14 (77.8%) were malignant; SMI correctly identified 12/14 malignant and 3/4 benign cases. **Conclusions**: SMI shows high diagnostic accuracy and reproducibility in the assessment of complex renal cystic lesions, with strong concordance to CEUS within the Bosniak 2019 system. By providing vascular characterization without contrast administration, SMI supports more personalized renal cyst management, enabling safer imaging for patients at risk from contrast agents and potentially reducing unnecessary interventions. Further multicenter validation is warranted to define its integration into precision-oriented multiparametric renal ultrasound protocols.

## 1. Introduction

Renal cystic lesions are increasingly detected as incidental findings due to the widespread use of cross-sectional imaging [1,2,3,4]. Accurate characterization of these lesions is crucial, as it directly influences patient management, surveillance intervals, and surgical decision-making [5,6]. The Bosniak classification system, first proposed in 1986 and most recently revised in 2019 [7], remains the cornerstone for stratifying the risk of malignancy in cystic renal masses [8,9,10]. This system relies primarily on imaging-based assessment of septa, wall thickness, calcifications, and the presence or absence of enhancement—a surrogate marker of vascularity and, consequently, of potential neoplastic transformation.

In this context, Superb Microvascular Imaging (SMI) is a contrast-free microvascular flow imaging technique that applies advanced clutter suppression and high-frame-rate Doppler processing to selectively display low-velocity blood flow in small-caliber vessels, while minimizing background tissue motion. Unlike conventional color and power Doppler, which are mainly sensitive to larger vessels and higher flow velocities, SMI is specifically optimized for depicting slow flow within thin walls and septa [11,12,13,14,15,16,17]. Several studies in different organs (including thyroid, breast, liver and renal lesions) have shown that SMI or similar microvascular imaging techniques increase the conspicuity of tumor neovascularization and correlate with histopathologic vascular patterns or contrast-enhanced imaging findings [18,19,20,21,22]. Nevertheless, its application in routine clinical practice remains limited, mainly due to its high operator dependency and restricted availability in non-specialized centers [23,24]. CEUS therefore represents a powerful tool in the integrated imaging assessment of renal lesions, although its diagnostic performance largely depends on the operator’s experience [25,26,27,28].

In this context, Superb Microvascular Imaging (SMI) represents a significant technical advancement in Doppler ultrasound [29,30,31,32]. By applying adaptive clutter suppression and high-frame-rate algorithms, SMI isolates low-velocity flow signals from motion artifacts, enabling visualization of microvascular structures down to the capillary level without the need for contrast media [33,34,35,36]. Preliminary evidence from hepatic, thyroid, and renal applications has shown promising correlation between SMI vascular patterns and histopathologic neovascularization [37,38,39,40].

Despite these encouraging results, the role of SMI in the standardized evaluation of cystic renal lesions remains underexplored. Specifically, few studies have investigated its concordance with CEUS in the context of the Bosniak classification. Furthermore, data on real-world applicability are still limited [41,42,43,44].

Therefore, the present retrospective study was designed to evaluate the diagnostic performance and inter-reader agreement of SMI for the 2019 Bosniak classification of cystic renal lesions, using CEUS as the reference standard for microvascular assessment. Conventional color and power Doppler were performed as part of the routine baseline ultrasound examination, but their findings were not prospectively recorded or analyzed as formal comparators, because the study was specifically focused on contrast-free microvascular flow imaging with SMI. The primary objective was to assess the concordance between SMI- and CEUS-based Bosniak categories and the ability of SMI to discriminate low- versus high-risk cystic lesions. Secondary objectives were to evaluate inter- and intra-reader agreement and to explore histopathologic correlation in surgically resected Bosniak III–IV lesions. Ultimately, we aimed to explore whether SMI could serve as a reliable, contrast-free adjunct for initial lesion characterization and follow-up, particularly in patients unsuitable for contrast administration, within a contemporary Bosniak-based diagnostic workflow.

## 2. Materials and Methods

The study protocol was approved by the local ethics committee at the University Hospital “Luigi Vanvitelli” (Naples, Italy), Prot. 4521/i/2025.

Written informed consent for the retrospective use of imaging and clinical data was obtained from all participants. All procedures were conducted in accordance with the principles of the Declaration of Helsinki and its subsequent amendments.

### 2.1. Study Design and Population

This retrospective observational study included 80 consecutive patients with kidney complex cysts, identified through ultrasound, CT or MRI, who subsequently underwent both US, with SMI integration, and CEUS examinations for further characterization spanning from January 2024 and July 2025. Inclusion criteria were (1) cystic renal lesion detectable by baseline ultrasound; (2) adequate acoustic window; and (3) completion of both SMI and CEUS studies. Exclusion criteria comprised, allergy to contrast media, pregnancy, or incomplete imaging data.

### 2.2. Imaging Protocol

Baseline B-mode and conventional ultrasound (CUS) examinations were performed by a radiologist with over ten years of experience in urological imaging. CUS was used to evaluate lesion location, size, shape, margins, echogenicity, and its relationship with the surrounding renal parenchyma. The optimal scan plane encompassing the lesion and adjacent cortex was selected to ensure consistency across modalities. All examinations were conducted on a Canon Aplio A550 ultrasound system (Canon Medical Systems, Europe) equipped with a convex array transducer operating at 1–6 MHz.

Following baseline assessment, Superb Microvascular Imaging (SMI) was performed in monochrome mode, with frame rate, gain, and dynamic range optimized for detecting slow-flow microvascular signals. Particular attention was given to septal and mural vascularization, key features in the Bosniak classification. Depth and focal settings were adapted to the lesion size and position to minimize motion artifacts.

Subsequently, each patient underwent Contrast-Enhanced Ultrasound (CEUS) in the same session after intravenous administration of 1.6 mL of SonoVue^®^ (Bracco Imaging, Milan, Italy) followed by a 5 mL saline flush. CEUS was performed using a very low mechanical index (MI < 0.1) to preserve microbubble stability, and real-time imaging was recorded for at least three minutes to cover arterial, parenchymal, and late phases. Additional views were obtained as needed to optimize lesion visualization.

### 2.3. Imaging Interpretation and Classification

All images were stored in the local Picture Archiving and Communication System (PACS), anonymized, and independently reviewed by two radiologists: one senior expert with over 15 years of experience in urological diagnostic imaging and one junior radiologist with fewer than 7 years of experience. Lesions were classified according to the 2019 Bosniak criteria, considering septal number and thickness, wall irregularity, and the presence or absence of enhancement or detectable microvascular flow on SMI. For each lesion, the Bosniak category determined by SMI was compared with that obtained from CEUS, which served as the reference standard.

### 2.4. Statistical Analysis

Statistical analysis was performed using IBM SPSS Statistics (version 27.0; IBM Corp., Chicago, IL, USA). Continuous variables were expressed as mean ± standard deviation and categorical variables as frequencies and percentages. Descriptive statistics were used to summarize patient demographics, lesion characteristics, and Bosniak classifications obtained with SMI and CEUS. To evaluate diagnostic concordance between the two techniques, Cohen’s kappa (κ) coefficient with 95% confidence intervals (CI) was calculated. Kappa values were interpreted according to the Landis and Koch scale: poor (<0.20), fair (0.21–0.40), moderate (0.41–0.60), good (0.61–0.80), and excellent (>0.81) agreement. For each Bosniak category, cross-tabulation of SMI versus CEUS results was performed, and McNemar’s test was used to identify significant differences in classification proportions. Spearman’s rank correlation was employed to assess ordinal association across Bosniak classes between SMI and CEUS. To determine the diagnostic performance of SMI in discriminating between low-risk (Bosniak I–IIF) and high-risk (Bosniak III–IV) lesions, Receiver Operating Characteristic (ROC) analysis was carried out, using CEUS as the reference standard. The area under the ROC curve (AUC), along with 95% CI, was calculated to estimate discriminative ability. Sensitivity, specificity, positive predictive value (PPV), and negative predictive value (NPV) were derived from the optimal cut-off point of the ROC curve using the Youden index. Finally, intra-reader reproducibility was evaluated in a subset of 20 randomly selected lesions re-analyzed after four weeks by the same observers, with intra-class correlation coefficients (ICC) computed to assess repeatability. 

A *p*-value < 0.05 was considered statistically significant for all tests.

## 3. Results

### 3.1. Patient and Lesion Characteristics

Eighty patients (46 men and 34 women; mean age 63 ± 11 years, range 41–84 years) were included in the analysis. A total of 92 renal cystic lesions were evaluated, as 12 patients presented with multiple lesions. Lesions were evenly distributed between the right (*n* = 47) and left (*n* = 45) kidneys, with no significant side predominance (*p* = 0.72). Mean lesion diameter was 2.6 ± 0.9 cm (range 1.0–4.8 cm). According to CEUS, lesions were categorized as follows: Bosniak I (10.8%), II (16.3%), IIF (28.2%), III (26.1%), and IV (18.6%). No complications or adverse events related to the use of SonoVue^®^ were recorded.

### 3.2. Concordance Between SMI and CEUS

**Figure 1:**
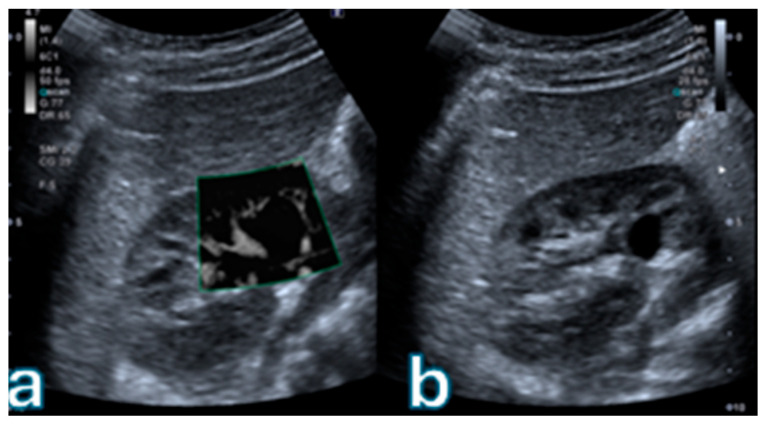
(**a**) Superb Microvascular Imaging (SMI) of a renal cyst demonstrates no detectable microvascular flow within the thin cyst wall, with absence of septa or mural nodules. (**b**) Corresponding B-mode ultrasound image shows an anechoic lesion with smooth margins and posterior acoustic enhancement, without any solid components. Overall features are consistent with a simple renal cyst (Bosniak I) according to the 2019 Bosniak classification.

Overall concordance between SMI and CEUS classifications was 80.4% (74 out of 92 lesions). Agreement was perfect for Bosniak I (100%) and II (100%), good for IIF (85%), and moderate for III (68%) and IV (64%) categories. Figure 1 illustrates a representative case of a simple cyst (Bosniak I) with no detectable microvascular flow.

**Figure 2:**
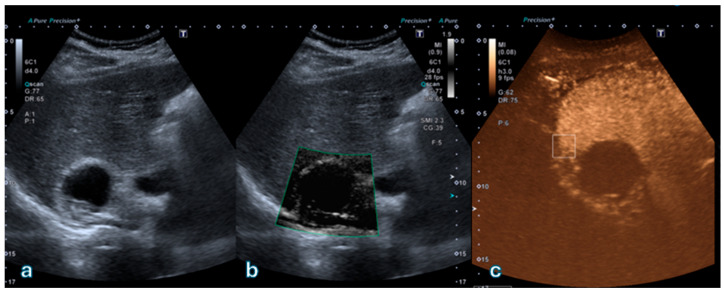
Complex cystic lesion located in the upper pole of the right kidney. (**a**) B-mode ultrasound shows a cyst with a dependent hyperechoic intracystic component movable with changes in patient position, suggesting proteinaceous or hemorrhagic material rather than a solid nodule. (**b**) Superb Microvascular Imaging (SMI) demonstrates no detectable vascular signal within the hyperechoic component or cyst wall. (**c**) Corresponding contrast-enhanced ultrasound (CEUS) image confirms absence of enhancement, consistent with a proteinaceous cyst classified as Bosniak II. This case exemplifies concordant findings between SMI and CEUS in a Bosniak II lesion.

Figure 2 shows a representative case of a Bosniak II cyst with an intracystic hyperechoic component lacking vascularization on both SMI and CEUS, confirming the benign nature of the lesion.

**Figure 3:**
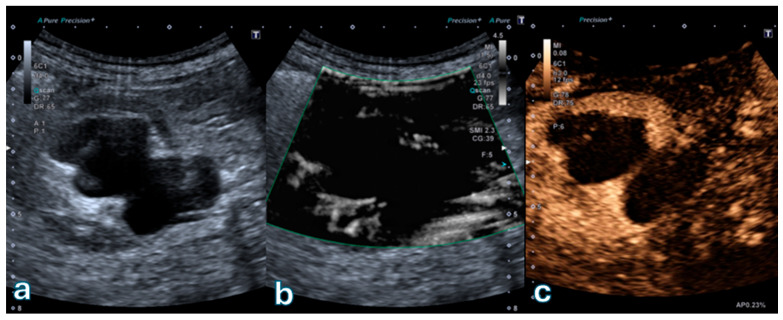
Complex renal cyst classified as Bosniak III. (**a**) B-mode ultrasound shows a multiloculated cystic lesion with thick, irregular walls. (**b**) Superb Microvascular Imaging (SMI) reveals intense microvascular signals along the thickened septa and walls, indicating marked vascularization. (**c**) Contrast-enhanced ultrasound (CEUS) demonstrates clear enhancement of the thick walls without a solid mural nodule, consistent with a Bosniak III indeterminate high-risk cystic lesion.

Figure 3 illustrates a representative Bosniak III cystic lesion with thickened, irregular septa. Superb Microvascular Imaging demonstrates discrete but definite microvascular signals within the septa, while CEUS confirms measurable septal enhancement in the absence of a solid mural nodule, consistent with an indeterminate high-risk cystic lesion.

**Figure 4:**
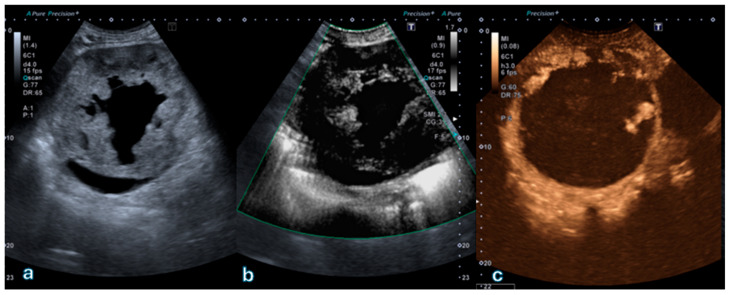
Partially cystic renal lesion with an eccentrically located, heterogeneously hyperechoic solid component. (**a**) B-mode ultrasound shows a mixed cystic–solid lesion with irregular internal architecture. (**b**) Superb Microvascular Imaging (SMI) demonstrates distinct microvascular flow signals within the solid component, indicating true vascularity. (**c**) Corresponding contrast-enhanced ultrasound (CEUS) image confirms contrast enhancement of the same solid area, consistent with a Bosniak IV lesion according to the 2019 Bosniak classification. This case exemplifies complete concordance between SMI and CEUS in detecting vascularized mural components suggestive of malignancy.

Figure 4 depicts a Bosniak IV cystic lesion characterized by a vascularized solid component visible on both SMI and CEUS, confirming their concordant diagnostic performance in detecting solid enhancing tissue.

**Figure 5:**
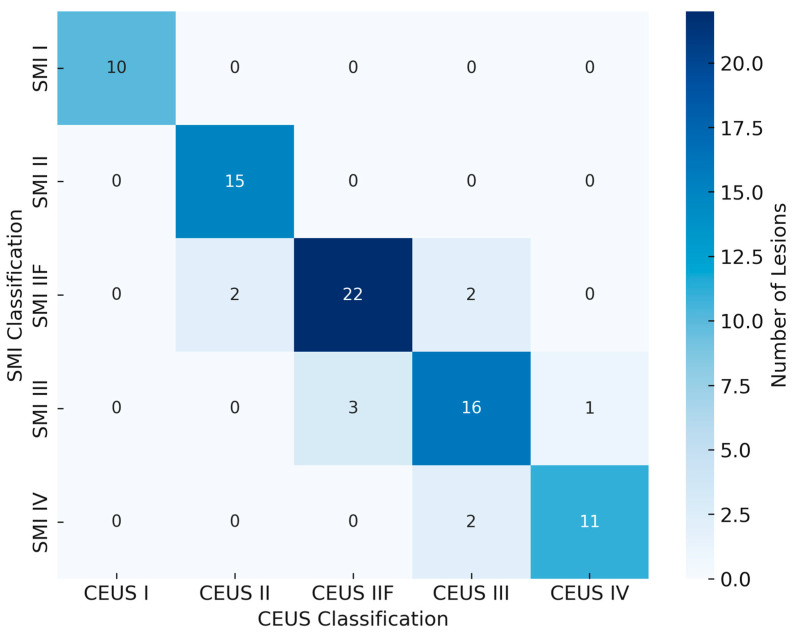
Heat map illustrating the agreement between SMI and CEUS classifications according to the 2019 Bosniak system. High concordance was observed for Bosniak I–IIF categories, while minor discrepancies occurred between IIF and III lesions.

Cross-tabulation analysis demonstrated that discrepancies mainly occurred between IIF and III categories, where subtle septal enhancement was variably detected. The distribution of SMI and CEUS classifications is illustrated in Figure 5.

**Figure 6:**
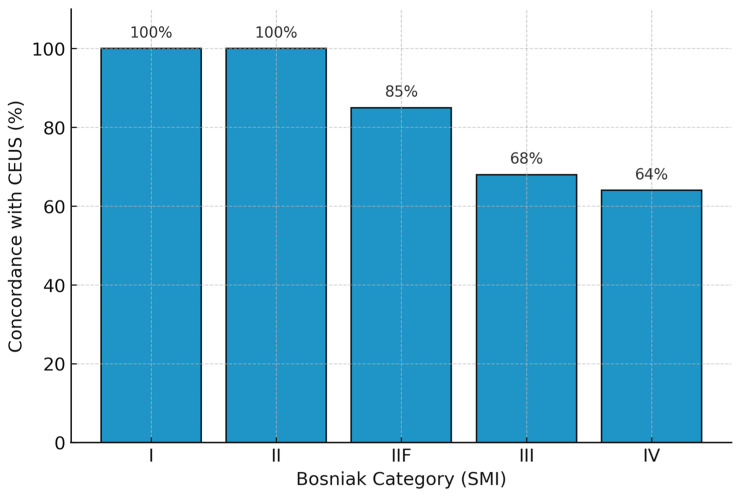
Bar chart showing SMI–CEUS concordance rates across Bosniak categories. Agreement was perfect for Bosniak I–II lesions (100%), good for IIF (85%), and moderate for III (68%) and IV (64%) categories.

Concordance rates for each Bosniak category are summarized in Figure 6, illustrating the progressive decrease in agreement from simple to complex cysts.

McNemar’s test revealed no statistically significant systematic difference between SMI and CEUS classifications (*p* = 0.41). Overall concordance between SMI and CEUS classifications was 83.7% (Table 1), with excellent diagnostic performance metrics as summarized in Table 2.

### 3.3. Diagnostic Performance of SMI

**Figure 7:**
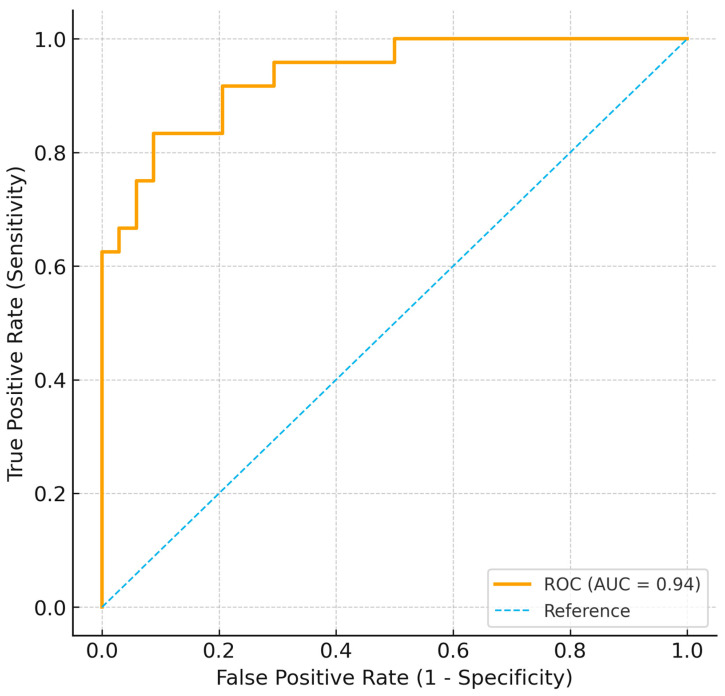
Receiver operating characteristic (ROC) curve showing the diagnostic performance of SMI for differentiating low- (Bosniak I–IIF) from high-risk (Bosniak III–IV) lesions using CEUS as the reference. The area under the curve (AUC) was 0.94, indicating excellent discrimination.

When CEUS was considered the reference standard, SMI correctly classified 77 out of 92 lesions, yielding a sensitivity of 88.5%, specificity of 90.0%, positive predictive value (PPV) of 89.7%, and negative predictive value (NPV) of 88.9% for identifying high-risk lesions (Bosniak III–IV). The Receiver Operating Characteristic (ROC) analysis demonstrated an area under the curve (AUC) of 0.94 (95% CI: 0.89–0.98), confirming excellent discriminative ability between low- and high-risk lesions (Figure 7).

### 3.4. Inter-Reader and Intra-Reader Reproducibility

The intra-class correlation coefficients (ICC) demonstrated excellent overall repeatability for SMI (ICC = 0.91) and CEUS (ICC = 0.94). However, diagnostic agreement was consistently higher for the senior radiologist compared with the junior reader (κ = 0.88 vs. 0.76 for SMI; κ = 0.91 vs. 0.82 for CEUS). These results suggest that both SMI and CEUS are highly reproducible imaging techniques, yet their diagnostic performance improves with reader experience.

### 3.5. Histopathological Correlation

Among the 24 Bosniak III–IV lesions, 18 were surgically resected and underwent histopathologic examination. Among the 18 resected Bosniak III–IV lesions, histopathologic examination confirmed malignancy in 14 cases (77.8%), with overall imaging concordance between SMI and CEUS of 88.9% (Table 3). Of these, 14 (77.8%) were confirmed as malignant—10 clear cell renal cell carcinomas, 2 papillary RCCs, and 2 multilocular cystic neoplasms of low malignant potential. The remaining 4 lesions (22.2%) were benign, including inflammatory cysts and hemorrhagic pseudocysts. The corresponding SMI classifications correctly identified 12 out of 14 malignant lesions (sensitivity 85.7%) and 3 out of 4 benign lesions (specificity 75.0%) relative to histopathologic outcome. On qualitative review, the benign lesions exhibited imaging features overlapping with those of malignant high-risk cysts: on CEUS they showed thick, enhancing walls and/or septa with corresponding SMI microvascular signals, thereby fulfilling Bosniak III–IV criteria and justifying surgical referral. Malignant lesions more frequently displayed eccentric mural nodules or more heterogeneous enhancement, but the small number of benign resected cases and the substantial overlap in appearances did not allow us to identify a reliable imaging pattern capable of distinguishing them preoperatively.

## 4. Discussion

This study evaluated the diagnostic performance of SMI in the assessment of complex renal cystic lesions, comparing it with CEUS as the reference technique within the framework of the 2019 Bosniak classification. In a cohort of 80 patients encompassing 92 renal cystic lesions, SMI demonstrated excellent concordance with CEUS (κ = 0.84) and a high discriminatory capacity (AUC = 0.94) between low-risk (Bosniak I–IIF) and high-risk (Bosniak III–IV) lesions. These results confirm that SMI is capable of accurately depicting septal and mural vascularization—key features in the Bosniak system—and suggest that it can serve as a reliable, non-contrast alternative for initial evaluation and follow-up.

The strong agreement observed between SMI and CEUS supports the notion that SMI can effectively detect slow-flow microvascular signals within cystic structures, allowing distinction between benign and potentially malignant lesions [45,46]. The absence of SMI vascular signal was consistently associated with the lack of contrast enhancement on CEUS, reinforcing the idea that avascular cysts can be confidently identified without the need for contrast administration [47,48,49]. In particular, SMI showed perfect concordance for Bosniak I and II lesions and good performance for IIF lesions, categories that represent the most clinically relevant group for surveillance. The sensitivity (88.5%) and specificity (90%) observed for SMI in identifying high-risk lesions highlight its solid diagnostic potential.

These findings are consistent with previous reports investigating SMI or similar microvascular flow imaging techniques. Mu et al. [42] demonstrated that SMI achieved an AUC of 0.869 in the Bosniak classification, while Leong et al. [50] observed improved vascular detection compared with conventional Doppler. Likewise, Járay et al. (2023) [17] reported that microvascular flow imaging enhanced the visualization of subtle vascular patterns in renal lesions, providing better differentiation of malignant cases. Collectively, these data support the emerging role of SMI as a non-invasive method for evaluating renal vascularity. The present study extends this evidence to cystic renal lesions, confirming that SMI correlates strongly with CEUS findings and can replicate its diagnostic outcomes in most scenarios.

From a methodological standpoint, this study adds several elements to the existing literature on SMI. First, it applies SMI within the framework of the 2019 Bosniak classification to a consecutive cohort of cystic renal lesions, using CEUS as a microvascular reference. Second, it provides quantitative diagnostic performance metrics, including ROC analysis, for the discrimination between low- and high-risk Bosniak categories. Third, it evaluates inter- and intra-reader agreement for both SMI and CEUS, highlighting the impact of reader experience. Finally, in the subset of surgically resected Bosniak III–IV lesions, it correlates SMI and CEUS findings with histopathology. Together, these aspects help to position SMI within contemporary Bosniak-based workflows rather than as an isolated imaging tool.

An additional observation derived from our experience concerns the influence of lesion complexity on SMI performance: concordance with CEUS was perfect for Bosniak I–II lesions, good for IIF, and only moderate for Bosniak III–IV cysts, and this pattern was closely related to lesion size and field of view (FOV). This phenomenon may be explained by a reduction in SMI sampling sensitivity with increasing FOV, leading to a lower ability to detect weak microvascular signals in peripheral septa or mural regions. This intrinsic limitation of SMI technology suggests that acquisition parameters (depth, gain, and frame rate) should be optimized according to lesion size to maintain adequate diagnostic sensitivity even in larger cysts. Another technical issue is the potential for SMI artifacts. In some published reports, any SMI signal has been interpreted as evidence of a vessel, which risks overestimating true vascularization. In our study, SMI signals were regarded as relevant only when they followed an anatomically plausible course, were reproducible over consecutive frames, and corresponded to a structure visible on B-mode images. Signals confined to the diaphragm, pleura or near-field, or strictly related to respiratory motion, were disregarded as artefactual. Nevertheless, residual misclassification cannot be entirely excluded, and accurate recognition of SMI artifacts remains an important limitation that requires specific training and standardization.

From a clinical standpoint, our findings suggest that SMI may be a useful contrast-free adjunct in the diagnostic workup and follow-up of cystic renal lesions, particularly in patients for whom contrast administration is undesirable. In Bosniak I and II cysts, the absence of SMI-detected vascularity was consistently associated with lack of enhancement on CEUS, which may help reassure benignity in appropriate clinical contexts. In Bosniak IIF lesions, SMI could potentially be used for surveillance to detect early changes in septal or mural vascularization, although this role requires confirmation in prospective studies. In more complex lesions (Bosniak III–IV), SMI contributed additional morphological and vascular information but did not replace CEUS, CT or MRI, which remain essential for preoperative planning and comprehensive characterization. Owing to its non-invasive nature, cost-effectiveness, and immediate availability, CEUS is particularly suited for outpatient settings and for centers aiming to streamline diagnostic workflows, and it offers a safe and reliable alternative for patients with renal insufficiency, in whom contrast-enhanced CT or MRI may be contraindicated. 

In our cohort, however, MRI was not systematically performed, and the present study was not designed to directly compare SMI with MRI; CEUS was adopted as the ultrasound reference technique, and any inference on the relative performance of SMI versus MRI is therefore indirect and should be interpreted with caution.

Another relevant aspect concerns the use of SMI and CEUS in patients with chronic kidney disease (CKD). CKD is associated with diffuse parenchymal changes, including cortical thinning, increased echogenicity and alterations in renal micro- and macrocirculation, which may potentially affect ultrasound depiction of both the parenchyma and cystic lesions. Under these conditions, CKD-related changes did not preclude the assessment of contrast uptake or microvascular signals within cyst walls and septa, and all lesions could be assigned a Bosniak category based on SMI and CEUS findings.

## 5. Limitations

This study has several limitations. First, its single-center, retrospective design and the relatively small sample size (80 patients and 92 cystic lesions) limit the generalizability of our findings, even though the study was sufficiently powered for the main statistical analyses. Second, only Bosniak III and IV lesions underwent histopathologic confirmation, whereas lower-category cysts were verified through imaging follow-up alone, which may introduce verification bias. Third, all examinations were performed using the same ultrasound system (Canon Aplio A550) and by radiologists experienced in CEUS and SMI interpretation, conditions that may not fully reflect variability in equipment and expertise across different centers.

Another relevant aspect emerging from our data concerns the influence of operator expertise on diagnostic reproducibility. Although both SMI and CEUS showed excellent intra-reader agreement, concordance values were consistently higher for the senior radiologist. This finding indicates that while these techniques are reliable tools for the initial discrimination between low- and high-risk cystic lesions, their accurate interpretation requires advanced familiarity with vascular imaging patterns and Bosniak criteria. Consequently, SMI and CEUS are best suited for high-volume or referral centers with dedicated expertise in urological imaging, where consistent acquisition parameters and interpretative experience can maximize their diagnostic potential.

Future research should aim to validate these findings across larger and more diverse populations, ideally within multicenter prospective trials. Quantitative SMI parameters, such as vascular index or vessel density, could be explored to provide objective criteria for lesion characterization. The establishment of standardized acquisition protocols and clear thresholds for vascular signal detection will be critical to ensure reproducibility and comparability across different institutions.

In summary, this single-center study suggests that SMI is a reproducible technique for evaluating cystic renal lesions, with diagnostic performance comparable to CEUS for Bosniak 2019 classification in our cohort. CEUS and cross-sectional contrast-enhanced imaging remain the reference modalities for complex cysts and preoperative assessment, and the role of SMI should currently be regarded as complementary rather than substitutive. Larger multicenter studies, ideally including standardized acquisition protocols and quantitative SMI parameters, are needed to confirm these findings and to better define how SMI can be integrated into multiparametric renal ultrasound in routine clinical practice.

## 6. Conclusions

Superb Microvascular Imaging (SMI) demonstrated high diagnostic accuracy and reproducibility in assessing complex renal cystic lesions, showing excellent concordance with Contrast-Enhanced Ultrasound (CEUS) according to the 2019 Bosniak classification. 

SMI accurately detected septal and mural vascularization, enabling reliable differentiation between low- and high-risk lesions without the need for contrast administration. These results support the use of SMI as a first-line, contrast-free imaging tool for diagnosis and follow-up, while CEUS and cross-sectional contrast imaging remain essential for complex or surgically relevant cases.

With further validation and standardization, SMI could become an integral component of multiparametric renal ultrasound.

## Figures and Tables

**Table 1:** Concordance between SMI and CEUS according to the 2019 Bosniak classification (n = 92 lesions). Distribution of cystic renal lesions according to the Bosniak 2019 classification on SMI and corresponding CEUS confirmation. Overall agreement between SMI and CEUS classifications was 83.7% (κ = 0.84, 95% CI 0.76–0.91). Discrepancies mainly occurred between Bosniak IIF and III categories.

Bosniak Category (SMI)	Number of Cystic Lesions (n)	Classification Confirmed by CEUS (n)	Concordance (%)
Bosniak I	10	10	100%
Bosniak II	15	15	100%
Bosniak IIF	26	22	85%
Bosniak III	24	16	68%
Bosniak IV	17	11	64%
Total	92	74	80.4%

**Table 2:** Diagnostic performance of SMI in comparison with CEUS for Bosniak classification. Diagnostic accuracy metrics of Superb Microvascular Imaging (SMI) compared with Contrast-Enhanced Ultrasound (CEUS) for renal cystic lesion classification according to Bosniak 2019. CEUS served as the reference standard. AUC = area under the ROC curve; ICC = intraclass correlation coefficient.

Parameter	Value	95% Confidence Interval	Interpretation
Sensitivity	88.5%	80.2–94.0%	High sensitivity for detection of high-risk (III–IV) lesions
Specificity	90.0%	81.9–95.3%	High ability to exclude low-risk lesions
Positive Predictive Value (PPV)	89.7%	81.0–94.8%	-
Negative Predictive Value (NPV)	88.9%	79.6–94.2%	-
Overall accuracy	89.1%	82.5–93.9%	Excellent Diagnostic Accuracy
Cohen’s κ (SMI vs. CEUS)	0.84	0.76–0.91	Excellent inter-modality agreement
AUC (ROC analysis)	0.94	0.89–0.98	Excellent discrimination between low- and high-risk lesions

**Table 3:** Histopathologic correlation of surgically resected Bosniak III–IV lesions (n = 18). Histopathologic outcomes of surgically resected Bosniak III–IV lesions and corresponding imaging classifications on SMI and CEUS. Malignant lesions included 10 clear cell RCCs, 2 papillary RCCs, and 2 multilocular cystic neoplasms. SMI correctly identified 12 of 14 malignant and 3 of 4 benign lesions, yielding sensitivity 85.7% and specificity 75.0% relative to histopathology.

Histopathologic Diagnosis	Number of Lesions (*n*)	SMI Classification (Bosniak 2019)	CEUS Classification (Bosniak 2019)	SMI–CEUS Concordance
Clear Cell Renal Cell Carcinoma	10	III (6), IV (4)	III (5), IV (5)	90%
Papillary Renal Cell Carcinoma	2	III (2)	III (2)	100%
Multilocular cystic renal neoplasm of low malignant potential	2	III (1), IV (1)	III (1), IV (1)	100%
Hemorrhagic or inflammatory cyst	3	IIF (2), III (1)	IIF (1), III (2)	67%
Simple cyst with fibrosis	1	II (1)	II (1)	100%
Total	18	-	-	88.9%

## Data Availability

The data presented in this study are available on request from the corresponding author for privacy reasons.
